# An investigation of the relation between tumor-to-liver ratio (TLR) and tumor-to-blood standard uptake ratio (SUR) in oncological FDG PET

**DOI:** 10.1186/s13550-016-0174-y

**Published:** 2016-03-02

**Authors:** Frank Hofheinz, Rebecca Bütof, Ivayla Apostolova, Klaus Zöphel, Ingo G. Steffen, Holger Amthauer, Jörg Kotzerke, Michael Baumann, Jörg van den Hoff

**Affiliations:** Helmholtz-Zentrum Dresden-Rossendorf, PET Center, Institute of Radiopharmaceutical Cancer Research, Bautzner Landstraße, Dresden, Germany; Department of Radiation Oncology, University Hospital Carl Gustav Carus, Technische Universität, Dresden, Germany; OncoRay - National Center for Radiation Research in Oncology, Dresden, Germany; Klinik für Radiologie und Nuklearmedizin, Universitätsklinikum Magdeburg A.ö.R., Magdeburg, Germany; Department of Nuclear Medicine, University Hospital Carl Gustav Carus, Technische Universität, Dresden, Germany; German Cancer Consortium (DKTK), Dresden, Germany; German Cancer Research Center (DKFZ), Heidelberg, Germany; Helmholtz-Zentrum Dresden-Rossendorf, Institute of Radiooncology, Dresden, Germany

**Keywords:** PET, FDG, Tumor-to-blood ratio, SUR, Tumor-to-liver ratio, TLR

## Abstract

**Background:**

The standardized uptake value (SUV) is the nearly exclusive means for quantitative evaluation of clinical [18F-]fluorodeoxyglucose (18F-FDG) positron emission tomography (PET) whole body investigations. However, the SUV methodology has well-known shortcomings. In this context, it has been recognized that at least part of the problems can be eliminated if tumor SUV is normalized to the SUV of a reference region in the liver (tumor-to-liver [TLR] ratio). In recent publications, we have systematically investigated the tumor-to-blood SUV ratio (SUR) for normalization of tumor SUVs which in our view offers principal advantages in comparison to TLR. The aim of this study was a comprehensive comparison of TLR and SUR in terms of quantification of tumor lesions.

**Methods:**

18F-FDG PET/CT was performed in 424 patients (557 scans) with different tumor entities prior to radio(chemo)therapy. In the PET images, SUV_max_ of the primary tumor was determined. SUV_liver_ was calculated in the inferior right lobe of the liver. SUV_blood_ was determined by manually delineating the aorta in the low-dose CT. TLR and SUR were computed and scan time corrected to 60 min p.i. (TLR_tc_ and SUR_tc_). Correlation analysis was performed for SUV_liver_ vs. SUV_blood_, TLR vs. SUR, SUV_liver_/SUV_blood_ vs. SUV_blood_,SUR_tc_/TLR vs. SUR_tc_, and SUR_tc_/TLR_tc_ vs. SUR_tc_. Variability of the respective ratios was assessed via histogram analysis. The prognostic value of TLR and TLR_tc_ for distant metastases-free survival (DM) was investigated with univariate Cox regression in a homogeneous subgroup (*N* = 130) and compared to previously published results for SUV and SUR_tc_.

**Results:**

Correlation analysis revealed a linear correlation of SUV_liver_ vs. SUV_blood_ (R ^2^=0.83) and of TLR vs. SUR_tc_ (*R*^2^=0.92). The SUV_liver_/SUV_blood_ ratio (mean ± s.d.) was 1.47 ± 0.18. For the SUR_tc_/TLR ratio, we obtained 1.14 ± 0.21 and for the SUR_tc_/TLR_tc_ ratio 1.38 ± 0.17. Survival analysis revealed TLR and TLR_tc_ as significant prognostic factors for DM (hazard ratio [HR] = 3.3 and HR = 3, respectively). Both hazard ratios are lower than that of SUR_tc_ (HR = 4.1) although this reduction does not reach statistical significance for the given limited group size. HRs of TLR and SUR_tc_ are both significantly higher than HR of SUV (HR = 2.2).

**Conclusions:**

Suitability of the liver as surrogate of arterial tracer supply for SUV normalization via TLR computation is limited. Further studies in sufficiently large patient groups are required to better characterize the relative performance of SUV, TLR, and SUR in different settings.

## Background

The standardized uptake value (SUV) currently is the nearly exclusive means for quantitative evaluation of clinical [18F]fluorodeoxyglucose (18F-FDG) positron emission tomography (PET) whole-body investigations. However, the SUV methodology has well-known shortcomings such as uptake time dependence of the SUV, unsatisfactory test/retest stability, susceptibility to errors in scanner calibration etc. [1–6] all of which adversely affect the reliability of the SUV as a surrogate of the metabolic rate of FDG (and ultimately of glucose consumption).

In this context, it has been recognized repeatedly that at least part of the mentioned problems can be reduced or eliminated if tumor SUV is normalized to the SUV of a suitable reference region [7]. Especially, the liver has drawn considerable attention as a useful reference region since the liver does not irreversibly trap the FDG and maintains a roughly constant SUV level during the time window relevant for whole-body FDG PET (about 60–120 min p.i.) [8–13]. In fact, the liver is the only reference region which so far has been studied and used extensively.

Using the tumor-to-liver-ratio (TLR) obviously removes some of the SUV limitations, i.e. possible inaccuracies regarding actually injected dose, scanner calibration, and patient weight index (either actual body weight, lean body mass [14], or body surface area [15]).

However, TLR exhibits an uptake time dependence comparable to that of tissue SUV itself (without a generally accepted means of quantitatively correcting for this effect in either case). Possibly more important, liver SUV (SUV_liver_) will exhibit an inter-individually (and possibly also intra-individually in case of aggressive treatment such as chemotherapy) variable relation to the given arterial tracer supply. On the other hand, it is the latter—expressed in SUV units (SUV_blood_)—which determines a given lesion’s observed SUV. Usefulness of the liver as a reference might be further compromised in the presence of liver disease or pharmacological intervention [16, 17]. Last but not least, depending on the investigation, the liver might simply not be routinely included in the field of view of the PET scan (e.g. at the participating sites in head and neck investigation, the liver is not always included in the FOV while a sufficiently large part of the aorta still is). For all these reasons, the liver cannot be considered an ideal reference region.

In recent publications, we have systematically investigated the tumor-to-blood SUV ratio (SUR) for normalization of tissue SUVs which in our view offers principal advantages in comparison to TLR. For one, the SUR approach by definition eliminates the influence of the persisting residual variability of SUV_blood_ on lesion SUV and ensures that SUR is superior to lesion SUV itself as a surrogate parameter of the metabolic rate of FDG [18]. Additionally, we were able to show that it is possible to reliably correct SUR for variations of the 18F-FDG uptake period under rather general and empirical well-fulfilled assumptions regarding the shape of the arterial input function (AIF) [19]. These advantageous properties of the SUR can be ultimately traced back to the empirical fact, that the AIF after FDG bolus injection exhibits an essentially invariant shape, following a simple inverse power law starting immediately after the bolus phase. Finally, we found strong evidence in a survival analysis of 130 patients with esophageal carcinoma that the superior properties of SUR also translate into a higher prognostic value [20]. While there is thus rather strong theoretical and empirical evidence for the superiority of SUR over SUV, it is so far an open question how performance of SUR compares to that of TLR.

The primary aim of the present investigation, therefore, was accurate determination of the degree of correlation between TLR and SUR. A secondary goal was to perform a first direct comparison of the performance of TLR and SUR as predictor of therapy outcome. For this purpose, we have utilized the patient group previously investigated in [20].

## Methods

### Patient group

In this retrospective study, 424 patients (358 men, 66 women) with mean age (range) 63 (37–85) years and different tumor entities (head and neck cancer *N* = 36 (HNC), non small cell lung cancer *N* = 178 (NSCLC), esophageal carcinoma *N* = 210 (EC)) were included. This patient group incorporates 130 patients with esophageal carcinoma treated with definitive radio(chemo)therapy previously investigated in the already mentioned study by Bütof et al. [20]. This subgroup is utilized in the present study for comparison of the prognostic value of TLR and SUR. In 84 out of 424 patients, two PET scans were performed at different days, where the first scan was before radio(chemo)therapy and the second scan afterwards. Time between first and second scan was on average 39.1 days (range 10–76). These data were included to study the intra-subject variability. In 49 out of 424 patients, dual time-point measurements were performed, and the respective late scans were included to extend the range of covered uptake times (up to 120 min). Altogether, 557 18F-FDG PET/CT scans were performed at University Hospital, Technische Universität Dresden (Site A) and at the University Hospital, Otto-von-Guericke University Magdeburg (Site B). Only scans where the liver as well as the aorta was in the FOV were included. Scan characteristics are summarized in Table 1. All scans besides the above mentioned were performed before radio(chemo)therapy and/or surgery. All patients had fasted for at least 6 h prior to 18F-FDG injection. The serum glucose concentration measured prior to injection was 5.9 mmol/L on average (range 3.3–10.7).

**Table 1:** Scan characteristics

	Site A	Site B
Number of patients	264	160
Number of scans	363	194
Scanner type	Biograph 16 PET/CT ^*a*^	Biograph mCT 64 ^*a*^
Dosage (MBq)	336 ± 38	234 ± 12
Scan start p.i. (min)	81 ± 15	81 ± 13
Range	51–116	55–120
Scan duration (min/per bed)	3	3
Reconstruction type iterations/subsets	OSEM 6i/4s	PSF+TOF 2i/21s

^*a*^Siemens Medical Solutions Inc., Knoxville, TN, USA

### Image analysis

ROI definition and ROI analyses were performed using the ROVER software, version 2.1.20 (ABX, Radeberg, Germany). Here and in the following, “ROI” is used synonymously with “VOI” for denoting a three-dimensional volume of interest.

The metabolically active part of the primary tumor was delineated by an automatic algorithm based on adaptive thresholding taking the local background into account [21]. The result of the automatic delineation was inspected visually by an experienced observer (one observer at each site) and corrected manually in case of obvious segmentation failure. For the resulting ROIs, SUV_max_ was computed. In the following, the index “max” is omitted, since only the maximum of lesion SUV and derived quantities (TLR, SUR) was considered in the evaluation.

The arterial blood SUV was determined by defining a roughly cylindrical aorta ROI in the attenuation CT data which than was transferred to the PET data. To exclude partial volume effects, a concentric safety margin was used in the transaxial planes, centering the ROI in the aorta. Planes showing high tracer uptake close to the aorta (pathological or otherwise) were excluded. The aorta ROI was positioned in the descending aorta, and the minimum volume was 5 ml. For the determination of the SUV_liver_, a spherical 3D ROI with a diameter of approximately 3 cm (14 ml) was placed on the normal inferior right lobe of the liver. TLR (SUR) was computed as ratio of maximum lesion SUV and mean SUV of the liver (aorta) ROI. In the following, we omit the index “mean” for liver (aorta) SUV. Scan time corrected SUR values were computed as described in [19]:

(1) $$ \begin{aligned} \text{SUR}_{\text{tc}} & = \frac{T_{0}}{T} \times \left({\text{SUR}} - V_{r}\right) +V_{r}\\ &= \frac{T_{0}}{T} \times {\text{SUR}} + \left(1 - \frac{T_{0}}{T} \right) \times V_{r}\,, \end{aligned}  $$

where *T* is the actual scan time p.i. and *T*_0_ is the chosen standard scan time to which the SURs are normalized (60 min in the present work). *V*_*r*_=0.53 ml/ml is an estimate of the apparent volume of distribution, corresponding to the *y*-axis intercept of a Patlak plot, previously derived in dynamic investigations [22]. Note, that for not too small SUR values, the influence of *V*_*r*_ is small and might be neglected, simplifying the correction formula to $\text {SUR}_{\text {tc}} = \frac {T_{0}}{T} \times \text {SUR}$.

As our previous work [20] demonstrates, the scan-time correction distinctly improves the prognostic value of the SUR, and it is thus the scan-time corrected value SUR_tc_ which should be compared against TLR. Of course, TLR is scan-time dependent as well but usually no attempt is made to correct for this effect, so the primarily relevant comparison is that between SUR_tc_ and this (scan time uncorrected) TLR. But, for completeness sake, we also compared SUR_tc_ with a scan-time-corrected TLR as follows. For scan time correction of TLR, we note that the SUV_liver_ is nearly time-independent in the relevant time window (≈ 60−120 min p.i.) so that the fractional change of TLR over time is essentially identical to the corresponding change of lesion SUV. In [19], we have demonstrated that scan time correction of lesion SUV is possible—although somewhat less accurate than for SUR—but in principle requires knowledge of SUV_blood_. However, an approximate correction is possible without this knowledge. When using the TLR approach instead of SUR (i.e. in absence of SUV_blood_ determination) this approximation would be the only feasible approach which we have thus used in the present investigation. The resulting correction formula is

(2) $$ \text{TLR}_{\mathrm tc} = \left(\frac{T_{0}}{T}\right)^{1-b} \times \text{TLR}  $$

where *b*=0.313 is a parameter describing the shape and decrease of the arterial input function over time (see [19] for details).

### Statistical analysis

Inter-subject variability of SUV_blood_ and SUV_liver_ was analyzed in the whole patient group where for patients with two PET scans only the first scan was used (*N* = 424). Inter-subject variability was assessed as standard deviation (SD) of the distribution of the respective SUV. Intra-subject variability of SUV_blood_ and SUV_liver_ was analyzed in the subgroup of 84 patients that received two scans on separate days. It was assessed as SD of the distribution of *Δ*SUV (= paired difference of the respective SUV in the second and first scan). Inter- and intra-subject variabilities were compared using a two-sided F test of the corresponding variances (squared SDs) testing the null hypothesis that they are equal.

Linear correlation analysis of liver vs. blood SUV and of TLR vs. SUR_tc_ (*N* = 557), respectively, was performed and visualized through scatterplots. Linear correlation analysis was also performed for LBR vs. SUV_blood_ as well as for SUR_tc_/TLR and SUR_tc_/TLR_tc_, respectively, vs. SUR_tc_. Variability of the respective ratios was assessed via histogram analysis and quantified by mean ± SD and 90 % confidence interval (CI).

Survival analysis was performed in the patient group already analyzed in [20] where the prognostic value of several PET parameters and of clinically relevant parameters for overall survival, locoregional tumor control, and distant metastases-free survival (DM) was investigated. In the present study, we investigate the prognostic value of TLR and TLR_tc_ for DM (for which the largest effect size was found in our previous study) using univariate Cox regression. For comparison, we also show the already published results for SUV and SUR_tc_. Hazard ratios were compared using the bootstrap method (random re-sampling with replacement; 10^5^ samples) to determine the statistical distribution of (HR_1_−HR_2_) from which the relevant *P* value than was derived. Statistical significance was assumed if *P*<0.05. Statistical analysis was performed with the *R language and environment for statistical computing* [23] version 3.1.2.

### Compliance with ethical standards

All procedures performed in studies involving human participants were in accordance with the ethical standards of the institutional and/or national research committee and with the 1964 Helsinki declaration and its later amendments or comparable ethical standards. Informed consent was obtained from all individual participants included in the study.

## Results

Voxel intensities of blood and liver ROIs exhibited comparable average standard devitations of 10.7 % (liver) and 11.3 % (blood), respectively, corresponding to standard errors of the mean values of 1.36 % (scan start p.i. 60–75 min: 1.22 %, >75 min 1.49 %) (liver) and 1.43 % (60–75 min 1.23 %, >75 min 1.60 %) (blood), respectively.

The mean values of SUV_blood_ and SUV_liver_ across all 424 investigated patients were 1.79 ± 0.36 and 2.56 ± 0.55, respectively. The mean intra-individual paired differences, *Δ*SUV_blood_ and *Δ*SUV_liver_, in 84 patients receiving two PET scans on different days were 0.05 ± 0.32 and 0.24 ± 0.42, respectively. This demonstrates that the inter- and intra-subject variability (i.e. the respective standard deviations) of both SUVs are of very similar magnitude (although the small positive difference between the inter- and intra-subject SUV_liver_ variability actually reaches statistical significance [ *P*=0.003]).

**Fig. 1:**
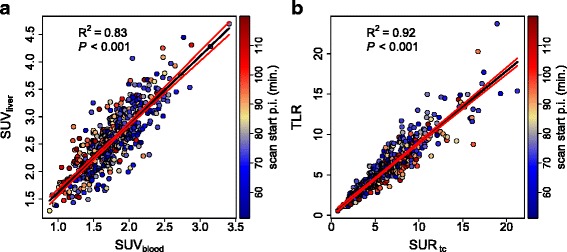
**a** Correlation between SUV_liver_ and SUV_blood_. **b** Correlation between TLR and SUR_tc_. *Black* lines represent the least squares straight line fits to the data. *Red* lines depict the 95 % CI

**Fig. 2:**
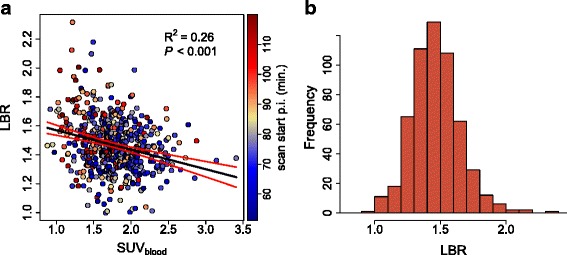
**a** Correlation between LBR and SUV_blood_. **b** Frequency distribution of LBR. **a** *Black* line represents the least squares straight line fit to the data. *Red* lines depict the 95 % CI

**Fig. 3:**
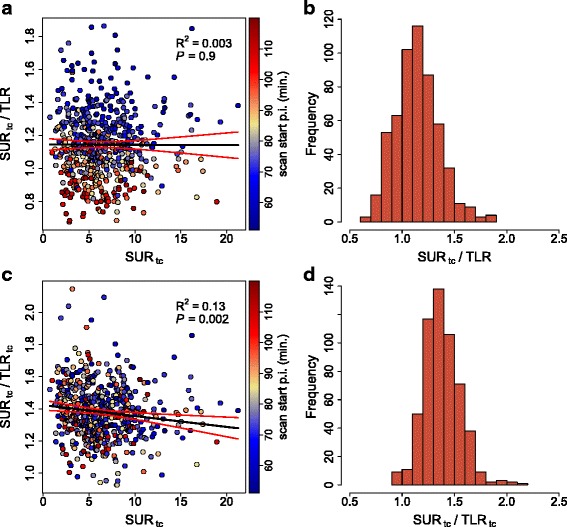
**a** Correlation between the SUR_tc_/TLR ratio and SUR_tc_. **b** Corresponding frequency distribution. **c** Correlation between the SUR_tc_/TLR_tc_ ratio and SUR_tc_. **d** Corresponding frequency distribution. *Black* lines represent the least squares straight line fits to the data. *Red* lines depict the 95 % CI

Correlation analysis revealed a pronounced linear correlation of SUV_liver_ and SUV_blood_ (*R*^2^=0.83) and of TLR and SUR_tc_ (*R*^2^=0.92). Corresponding scatterplots are shown in Fig. 1. There were no notable differences between investigating sites, tumor entities, or tumor size (Table 2). For LBR, we obtained 1.47 ± 0.18 (90 % CI 1.2–1.78). Corresponding scatterplot and histogram are shown in Fig. 2. For the SUR_tc_/TLR ratio, we obtained 1.14 ± 0.21 (90 % CI 0.82–1.48) and for the SUR_tc_/TLR_tc_ ratio 1.38 ± 0.17 (90 % CI 1.12–1.65). Corresponding scatterplots and histograms are shown in Fig. 3. Obviously, time correction of TLR reduces the fractional variability of the ratio (from about 18 to 12 %). For all ratios, there was no notable difference between investigating sites, tumor entities, or tumor size (Table 3).

**Table 2:** Correlation (*R* ^2^) of SUV_blood_ vs. SUV_liver_ and TLR vs. SUR. All correlations were significant (*P* < 0.001)

Group	Liver vs. blood	TLR vs. SUR_tc_
All	0.83	0.92
Site		
Site A	0.8	0.92
Site B	0.79	0.93
Tumor entity		
HNC	0.83	0.93
NSCLC	0.81	0.92
EC	0.77	0.92
Tumor size		
Volume <10 ml	0.81	0.92
Volume ≥10 ml	0.85	0.91

**Table 3:** Variability of the ratios LBR, SUR_tc_/TLR, and SUR_tc_/TLR_tc_

	LBR		SUR_tc_/TLR		SUR_tc_/TLR_tc_	
Group	Mean ± SD	90 % CI	Mean ± SD	90 % CI	Mean ± SD	90 % CI
All	1.47 ± 0.18	1.2–1.78	1.14 ± 0.21	0.82–1.48	1.38 ± 0.17	1.12–1.65
Site						
Site A	1.45 ± 0.19	1.18–1.78	1.14 ± 0.22	0.82–1.55	1.37 ± 0.18	1.11–1.66
Site B	1.5 ± 0.18	1.23–1.79	1.15 ± 0.18	0.84–1.43	1.4 ± 0.16	1.15–1.64
Tumor entity						
HNC	1.44 ± 0.15	1.2–1.66	1.16 ± 0.21	0.85–1.49	1.36 ± 0.14	1.12–1.61
NSCLC	1.49 ± 0.18	1.23–1.8	1.14 ± 0.18	0.82–1.43	1.39 ± 0.16	1.14–1.64
EC	1.45 ± 0.19	1.18–1.78	1.15 ± 0.23	0.81–1.57	1.37 ± 0.19	1.09–1.66
Tumor size						
Volume <10 ml	1.48 ± 0.19	1.18–1.78	1.16 ± 0.23	0.8–1.58	1.4 ± 0.19	1.12–1.66
Volume ≥ 10 ml	1.46 ± 0.18	1.2–1.78	1.13 ± 0.19	0.83–1.42	1.36 ± 0.16	1.13–1.62

**Fig. 4:**
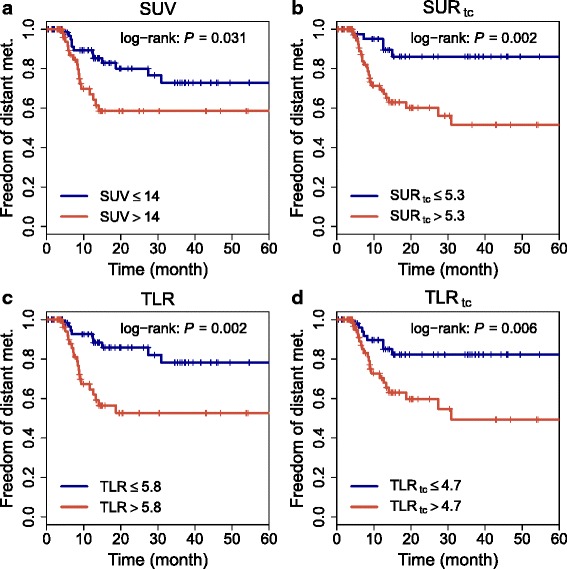
Kaplan-Meier curves with respect to DM (*N* = 130 patients with esophageal carcinoma). Results for SUV and SUR_tc_ have been taken from our paper [20]

Survival analysis (*N* = 130) revealed TLR and TLR_tc_ as significant prognostic factors for DM without being significantly different from each other (HR = 3.3 and HR = 3, respectively). These hazard ratios are to be compared with the previously reported results from this patient group [20] for SUV (HR = 2.2) and SUR_tc_ (HR = 4.1). Further details can be found in Table 4. Corresponding Kaplan-Meier curves are shown in Fig. 4.

**Table 4:** Univariate Cox regression with respect to DM (*N* = 130 patients with esophageal carcinoma)

Parameter	Risk	HR	95 % CI	*P* value
SUV	>14	2.2	1.1–4.7	0.035
TLR	>5.8	3.3	1.5–7.3	0.003
TLR_tc_	>4.7	3	1.3–6.7	0.009
SUR_tc_	>5.3	4.1	1.5–10.7	0.004

Results for SUV and SUR_tc_ have been taken from our paper [20]

According to bootstrap resampling, HRs of TLR and SUR_tc_ were both significantly higher than the HR of SUV (*P*=0.019 and *P*=0.048, respectively) while the HR difference between TLR_tc_ and SUV was not significant (*P*=0.17). The HR difference between SUR and TLR or TLR_tc_ was also not significant (*P*=0.31 or *P*=0.16).

## Discussion

Figure 1a demonstrates a pronounced but far from perfect linear correlation between SUV_liver_ and SUV_blood_. Indeed, a stronger correlation of both quantities might be expected since in any single investigation, tracer uptake at a given time point in any given target region (the liver included) is proportional to the overall scale of the AIF and, consequently, to its value at the chosen time point. Thus, in view of the fact that the AIF exhibits an essentially invariant shape across different investigations [18, 19] and presuming the metabolic state of the liver could be considered sufficiently similar with respect to uptake and release of FDG across different investigations/patients, a near-perfect linear correlation (actually, a proportionality) would result in Fig. 1a, at least for sufficiently standardized uptake time. However, this is not the case.

Considering the possible explanations, it is easily verified that the deviations from a perfect straight line are not a consequence of statistical errors due to the given signal to noise ratio of the corresponding ROI averages [24]. Systematic errors due to regionally variable accuracy of attenuation or scatter correction, too, would not be able to disturb the linear correlation to such an extent.

Excluding measurement-related effects, two obvious possible explanations remain for the sizable deviations from perfect linear correlation. First, the correlation might be adversely affected by differences in uptake time (color-coded in the scatter plots) since the time activity curves in liver and blood have different shapes and the LBR, thus, is time-dependent (slowly increasing over time). It is obvious from the color-coding of the data points according to uptake time in Figs. 1a and 2a that this effect at most is responsible for a minor part of the scatter, driving LBR to somewhat higher values at late times (which on average correspond to lower SUV_blood_ values, explaining the small but significant negative correlation of LBR and SUV_blood_ in Fig. 2a).

The only remaining plausible explanation in our view is to attribute the scatter to non-negligible inter- and intra-individual quantitative differences of FDG kinetics in the liver between different patients or scans.

Regarding the degree of intra-subject variability of SUV_liver_ and SUV_blood_ separately, our results are in good quantitative agreement with [4]. Our data furthermore demonstrate that inter-subject variability of both quantities is very similar to the respective intra-subject variability (although the difference reaches statistical significance in case of the liver where inter-subject variability is slightly larger than the intra-subject variability). We believe this to be an important observation in itself; intra- and inter-individual fluctuations of SUV_liver_ and SUV_blood_ do have very similar magnitude.

While our data thus essentially confirm and augment existing data regarding inter-scan variability of SUV_liver_ and SUV_blood_ they, furthermore, provide to our knowledge the first comprehensive investigation of the degree of correlation between both quantities. Regarding utilization of the liver as reference region for lesion SUV normalization, our data demonstrate that the liver in fact cannot be considered a highly accurate substitute for actual arterial tracer supply; from the data shown in Fig. 2, we derive an LBR of 1.47 ± 0.18 with a 90 % confidence interval of 1.2–1.78 whose limits differ by 48 %. These fluctuations directly translate into spurious fluctuations of the derived TLR values which would erroneously be interpreted as being due to changes in lesion metabolism.

The magnitude of this effect is demonstrated in Fig. 1b where TLR is compared to SUR_tc_. While the correlation coefficient is larger than that in Fig. 1a (ultimately a consequence of the much higher dynamic range of SUR and TLR in comparison to SUV_blood_ and SUV_liver_), the SUR_tc_/TLR ratio in fact exhibits a fractional variability that is distinctly higher than that of LBR (about 18 vs. 12 %) which also is apparent from a comparison of Fig. 2 and Fig. 3a, b. This increased variability is caused by the fact that we use SUR_tc_ here, rather than the scan-time uncorrected SUR for the reasons explained in the introduction.

Since uptake time correction of TLR is currently not applied in clinical routine, one thus actually faces a variability of TLR in comparison to SUR_tc_ of 1.14 ± 0.21 (90 % CI 0.82–1.48) if actual scan times are as variable as in our study group.

Also performing uptake time correction for TLR approximates the situation where uptake times would be strictly standardized (to 60 min in the present case). This leads to the results shown in Fig. 3 which indeed demonstrate a very similar mean and SD of the SUR_tc_/TLR_tc_ ratio in comparison to the LBR data in Fig. 2. This should be expected if the scan-time correction performs well since the time dependence of LBR itself is rather weak as already discussed above. The bottom line here is that the SUR_tc_/TLR ratio exhibits variability which is at least as large as that of LBR but will be substantially higher under typical clinical conditions where uptake times can vary considerably [25, 26].

Accepting our point of view that SUR_tc_ for principal reasons should be considered to represent the best available surrogate of lesion glucose consumption (since it uses the “correct” way of normalizing directly to the actual arterial tracer supply and accounts for time dependence of both; lesion uptake and AIF) the stated variability of SUR_tc_/TLR represents a principal limitation of the TLR as a surrogate of lesion glycolysis.

Of course, even if this conjecture is correct, the real question is how TLR performs in comparison to SUV and SUR regarding its prognostic value. In comparison to SUV, it has been repeatedly shown [12, 13, 27] that TLR is capable of improving the prognostic value of the PET investigation. It thus is unquestionably a valuable concept. On the other hand, the much more recently proposed SUR has not yet seen wide-spread evaluation and a comparison to TLR has been completely missing so far. We therefore consider the results presented in Fig. 4 and Table 4 of special interest. They clearly demonstrate that TLR as well as SUR_tc_ are superior to SUV as predictors of DM in the investigated patient group. Uptake time correction of TLR (TLR_tc_) did not improve the prognostic value as described by the hazard ratios in Table 4 in comparison to TLR. This was an initially somewhat unexpected result since uptake time correction reduces the deviations from a constant SUR_tc_/TLR_tc_ ratio. This finding might indicate that in our patient group the improved prognostic value of SUR_tc_ is caused mainly by the beneficial influence of normalization to SUV_blood_ rather than by scan-time correction. However, further investigations will be necessary to clarify this question.

The already previously reported that HR of SUR_tc_ is distinctly higher than HR of TLR (HR[ SUR_tc_] = 4.1, HR[TLR] = 3.3). In the given study group with its limited group size, though, the increase of HR is not large enough to reach statistical significance in the bootstrap resampling analysis. This indicates that the principal advantages of SUR_tc_ over TLR (consideration of actual arterial tracer supply and accurate uptake time correction) are not decisive at the given level of statistical accuracy available in our study group. Nevertheless, we believe that the observed very weak indication of superiority of SUR_tc_ over TLR is a sufficient incentive to further investigate the relative performance of TLR and SUR in other patient groups. Personally, we believe it very likely that ultimate superiority of SUR over TLR will be demonstrated since the latter parameter does not allow to fully account for the inter- and intra-individual variability of arterial tracer supply (and thus remains subject to spurious changes which are unrelated to differences in lesion glycolysis). In any case, both parameters are clearly superior to SUV and in practical terms might be viewed to some extent as complementary concepts (rather than competing ones) since the blood pool (aorta) will frequently be covered in the FOV even when the liver is not (or when the presence of liver disease precludes use of the TLR approach).

Overall, it seems worthwhile and promising to further investigate the relative performance of SUV, TLR, and SUR in other patient groups with the ultimate goal of deciding whether SUR can be considered as generally superior to SUV and TLR. If this turns out to be true, it would constitute a strong incentive to use SUR as a drop-in replacement for the current SUV and TLR methodology (or at least as an attractive alternative to the latter one) in clinical whole body FDG PET.

## Conclusions

Suitability of the liver as a surrogate of arterial tracer supply for SUV normalization via TLR computation is limited due to the less-than-perfect correlation between blood and liver SUV, and the SUR approach remains attractive for principal as well as practical reasons. Regarding their respective prognostic value, both, TLR and SUR significantly outperformed SUV. Further studies in sufficiently large patient groups are required to better characterize the relative performance of SUV, TLR, and SUR in different settings.

## References

[1] Hamberg LM, Hunter GJ, Alpert NM, Choi NC, Babich JW, Fischman AJ (1994). The dose uptake ratio as an index of glucose metabolism: useful parameter or oversimplification?. J Nucl Med.

[2] Keyes Jr JW (1995). Suv: Standard uptake or silly useless value?. J Nucl Med.

[3] Huang SC (2000). Anatomy of suv. Nucl Med Biol.

[4] Boktor RR, Walker G, Stacey R, Gledhill S, Pitman AG (2013). Reference range for intrapatient variability in blood-pool and liver suv for 18f-fdg pet. J Nucl Med.

[5] Weber WA, Gatsonis CA, Mozley PD, Hanna LG, Shields AF, Aberle DR, Govindan R, Torigan DA, Karp JS, Jian QY, et al.Repeatability of 18F-FDG PET/CT in advanced non-small cell lung cancer: prospective assessment in two multicenter trials. J Nucl Med. 2015:114–147728. 10.2967/jnumed.114.147728 PMC4699428 25908829

[6] Hristova I, Boellaard R, Vogel W, Mottaghy F, Marreaud S, Collette S, Schöffski P, Sanfilippo R, Dewji R, van der Graaf W (2015). Retrospective quality control review of fdg scans in the imaging sub-study of palette eortc 62072/veg110727: a randomized, double-blind, placebo-controlled phase iii trial. Eur J Nucl Med Mol Imaging.

[7] Schulte M, Brecht-Krauss D, Werner M, Hartwig E (1999). Evaluation of neoadjuvant therapy response of osteogenic sarcoma using fdg pet. J Nucl Med.

[8] Bares R, Klever P, Hauptmann S, Hellwig D, Fass J, Cremerius U, Schumpelick V, Mittermayer C, Büll U (1994). F-18 fluorodeoxyglucose pet in vivo evaluation of pancreatic glucose metabolism for detection of pancreatic cancer. Radiology.

[9] Delbeke D, Martin WH, Sandler MP, Chapman WC, Wright Jr JK, Pinson CW (1998). Evaluation of benign vs malignant hepatic lesions with positron emission tomography. Arch Surg.

[10] Flamen P, Van Cutsem E, Lerut A, Cambier JP, Haustermans K, Bormans G, De Leyn P, Van Raemdonck D, De Wever W, Ectors N (2002). Positron emission tomography for assessment of the response to induction radiochemotherapy in locally advanced oesophageal cancer. Ann Oncol.

[11] Shiono S, Abiko M, Okazaki T, Chiba M, Yabuki H, Sato T (2011). Positron emission tomography for predicting recurrence in stage I lung adenocarcinoma: standardized uptake value corrected by mean liver standardized uptake value. Eur J Cardiothorac Surg.

[12] Kunikowska J, Matyskiel R, Toutounchi S, Grabowska-Derlatka L, Koperski Ł, Królicki L (2014). What parameters from 18f-fdg pet/ct are useful in evaluation of adrenal lesions?. Eur J Nucl Med Mol Imaging.

[13] Bahce I, Vos C, Dickhoff C, Hartemink K, Dahele M, Smit E, Boellaard R, Hoekstra O, Thunnissen E (2014). Metabolic activity measured by fdg pet predicts pathological response in locally advanced superior sulcus nsclc. Lung Cancer.

[14] Sugawara Y, Zasadny KR, Neuhoff AW, Wahl RL (1999). Reevaluation of the standardized uptake value for fdg: variations with body weight and methods for correction. Radiology.

[15] Kim CK, Gupta NC, Chandramouli B, Alavi A (1994). Standardized uptake values of fdg: body surface area correction is preferable to body weight correction. J Nucl Med.

[16] Mueller M, Reimold M, Pfannenberg C, Bares R (2009). Influence of the acute phase reaction on the [18f] fdg uptake of the liver. J Nucl Med.

[17] Ceriani L, Suriano S, Ruberto T, Zucca E, Giovanella L (2012). 18f-fdg uptake changes in liver and mediastinum during chemotherapy in patients with diffuse large b-cell lymphoma. Clin Nucl Med.

[18] van den Hoff J, Oehme L, Schramm G, Maus J, Lougovski A, Petr J, Beuthien-Baumann B, Hofheinz F (2013). The pet-derived tumor-to-blood standard uptake ratio (sur) is superior to tumor suv as a surrogate parameter of the metabolic rate of fdg. EJNMMI Res.

[19] van den Hoff J, Lougovski A, Schramm G, Maus J, Oehme L, Petr J, Beuthien-Baumann B, Kotzerke J, Hofheinz F (2014). Correction of scan time dependence of standard uptake values in oncological pet. EJNMMI Res.

[20] Bütof R, Hofheinz F, Zöphel K, Stadelmann T, Schmollack J, Jentsch C, Löck S, Kotzerke J, Baumann M, van den Hoff J (2015). Prognostic value of pretherapeutic tumor-to-blood standardized uptake ratio in patients with esophageal carcinoma. J Nucl Med.

[21] Hofheinz F, Pötzsch C, Oehme L, Beuthien-Baumann B, Steinbach J, Kotzerke J, van den Hoff J (2012). Automatic volume delineation in oncological PET. Evaluation of a dedicated software tool and comparison with manual delineation in clinical data sets. Nuklearmedizin.

[22] van den Hoff J, Hofheinz F, Oehme L, Schramm G, Langner J, Beuthien-Baumann B, Steinbach J, Kotzerke J (2013). Dual time point based quantification of metabolic uptake rates in 18f-fdg pet. EJNMMI Res.

[23] R Core Team. R: a language and environment for statistical computing. Vienna, Austria, R Foundation for Statistical Computing; 2014. Vienna, Austria, R Foundation for Statistical Computing. http://www.R-project.org/.

[24] Lodge MA, Chaudhry MA, Wahl RL (2012). Noise considerations for pet quantification using maximum and peak standardized uptake value. J Nucl Med.

[25] Beyer T, Czernin J, Freudenberg LS (2011). Variations in clinical pet/ct operations: results of an international survey of active pet/ct users. J Nucl Med.

[26] Tahari AK, Wahl RL. Quantitative fdg pet/ct in the community: experience from interpretation of outside oncologic pet/ct exams in referred cancer patients. J Med Imaging Radiat Oncol. 2013. 10.1111/1754-9485.12140 PMC3975673 24314055

[27] Tournoy K, Maddens S, Gosselin R, Van Maele G, Van Meerbeeck J, Kelles A (2007). Integrated fdg-pet/ct does not make invasive staging of the intrathoracic lymph nodes in non-small cell lung cancer redundant: a prospective study. Thorax.

